# Coordination Complex Formation and Redox Properties of Kynurenic and Xanthurenic Acid Can Affect Brain Tissue Homeodynamics

**DOI:** 10.3390/antiox8100476

**Published:** 2019-10-11

**Authors:** Lenka Kubicova, Franz Hadacek, Gert Bachmann, Wolfram Weckwerth, Vladimir Chobot

**Affiliations:** 1 Division of Molecular Systems Biology, Department of Ecogenomics and Systems Biology, Faculty of Life Sciences, University of Vienna, Althanstrasse 14, A-1090 Vienna, Austria; lenka.kubicova@univie.ac.at (L.K.); gert.bachmann@univie.ac.at (G.B.); wolfram.weckwerth@univie.ac.at (W.W.); 2 Department of Plant Biochemistry, Albrecht-von-Haller Institut, Georg-August-Universität Göttingen, Justus-von-Liebig-Weg 11, D-37077 Göttingen, Germany; franz.hadacek@biologie.uni-goettingen.de; 3 Vienna Metabolomics Center (VIME), University of Vienna, Althanstrasse 14, 1090 Vienna, Austria

**Keywords:** Alzheimer’s disease, antioxidant, Fenton reaction, hydroxyl radical, iron chelates, kynurenines, neurodegeneration, Parkinsonism, reactive oxygen species

## Abstract

Reactive oxygen species (ROS) are known for their participation in various physiological and pathological processes in organisms, including ageing or degeneration. Kynurenine pathway metabolites, such as kynurenic (KYNA) or xanthurenic (XA) acid, can affect neurodegenerative diseases due to their ROS scavenging and Fe ion coordination complex formation but insights are still incomplete. Therefore, we investigated the formation and antioxidant capabilities of KYNA– and XA–Fe complexes by nano-electrospray−mass spectrometry, differential pulse voltammetry, deoxyribose degradation and Fe^II^ autoxidation assays. XA formed coordination complexes with Fe^II^ or Fe^III^ ions and was an effective antioxidant. By contrast, only Fe^II^–KYNA complexes could be detected. Moreover, KYNA showed no antioxidant effects in the FeCl_3_/ascorbic acid deoxyribose degradation assay variant and only negligible activities in the Fe^II^ autoxidation assay. Coordination complexes of Fe ions with KYNA probably stabilize KYNA in its keto tautomer form. Nevertheless, both KYNA and XA exhibited sufficient antioxidant activities in some of the employed assay variants. The results provide evidence that both have the potential to alleviate neurodegenerative diseases by helping to maintain tissue redox homeodynamics.

## 1. Introduction

**Figure 1:**
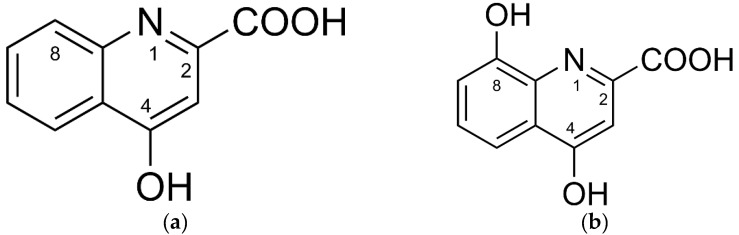
Chemical structures of (**a**) kynurenic acid and (**b**) xanthurenic acid.

Reactive oxygen species (ROS) are inherent to many cellular signal cascades in physiological and also pathological organismic processes [1,2] and cell reproduction [3]. Therefore, ROS concentration control is one of the key requirements that constrain efficient functioning of enzymatic and non-enzymatic systems in aerobic organisms [1,4]. The tryptophan metabolites kynurenic (KYNA) and xanthurenic (XA) acid represent low-molecular-weight components of the redox homeodynamics control system (Figure 1) [5].

KYNA and XA are kynurenine pathway-derived quinolines that, similarly to other kynurenines, can affect physiological and pathological processes of the central nervous, immune and vascular systems [6,7,8,9]. In general, KYNA and XA seem to play an important role in the mammalian nervous system, of which complete understanding is still lacking [6,10] despite KYNA’s reputation as a neuroprotective agent and an endogenous antioxidant [6,11]. Pyridoxine (vitamin B_6_) deficiency increases the concentrations of both acids in the brain tissue [12]. Contrary to KYNA, XA is able to penetrate the blood–brain barrier (BBB). Therefore, XA can be accumulated in the brain tissue (1 µM), either by endogenous biosynthesis or transport through the BBB [13]. The brain concentrations of KYNA are relatively low, about 150 nM [14]. However, brain concentrations of both acids can fluctuate depending on the metabolic situations [13,15]. Although KYNA penetrates only poorly through the BBB [11], exogenous KYNA, as a constituent of vegetables (e.g., potatoes) [16], may affect brain functions indirectly via positive effects on the gut−brain axis [17,18]. Furthermore, KYNA and its selected chemical derivatives can prolong longevity of bdelloid rotifers significantly [19].

By forming coordination complexes with transition metals, XA might affect degenerative diseases [5]. These metals, e.g., iron, accumulate in the brain. Ageing, neurodegenerative diseases in general and specifically damage of the blood−brain barrier can contribute to this process [20,21]. In healthy tissues, Fe ions are usually liganded by storage molecules. However, “poorly liganded” Fe ions are able to catalyze various chemical reactions that generate reactive oxygen species (ROS), especially cytotoxic hydroxyl radicals. In high concentrations, these ROS disturb the cellular redox homeodynamics and can trigger various pathological processes [21,22,23]. For example, the iron concentrations in senile plaques in brains of patients suffering from Alzheimer’s disease can increase up to 0.9 mM compared to the healthy controls of 0.3 mM [24].

Since KYNA and XA are phenolic molecules, they possess reducing capabilities that can affect ROS concentrations. Albeit that numerous studies have explored the coordination complex formation properties of KYNA and XA as well as their ROS scavenging activities, the reported results make it difficult to draw consistent conclusions. KYNA is a known antioxidant [5,25]. However, Minakana et al. found no antioxidant effects of KYNA in reaction mixtures with liver microsomes [26]. Furthermore, KYNA appeared to be weakly liganded by iron due to its keto-enol tautomery [26]. Similarly, XA also showed inconsistent results in terms of antioxidant activity. XA inhibited lipid peroxidation and protected NADP-isocitrate dehydrogenase against its deactivation by ROS [27]; it decreased oxidative damage of DNA [28]. Conversely, XA promoted oxidative deactivation of aconitase [29].

Therefore, in attempts to study the interplay of reducing ability, antioxidant effects and coordination complex formation of KYNA and XA with iron, this study investigated the coordination complex formation by nano-ESI−MS (nano-electrospray ionization−mass spectrometry). The changes in redox activity of the central atom and the ligands were characterized by differential pulse voltammetry, deoxyribose degradation and Fe^II^ autoxidation assays. This methodological approach was successful to explore ROS scavenging and coordination complex formation of other kynurenines with iron, such as anthranilic, 3-hydroxyanthranilic [30] and quinolinic acid [31,32].

## 2. Materials and Methods

### 2.1. Chemicals

All used chemicals were purchased from Sigma-Aldrich (Schnelldorf, Germany). Water was of Milli-Q quality (Milli-Q Advantage A10 System, Milllipore SAS, Molsheim, France).

### 2.2. Mass Spectrometry

Direct infusion nano-electrospray ionization mass spectrometry was carried out in positive ionization mode on a Thermo Electron LTQ-Orbitrap XL mass spectrometer equipped with a nano electrospray ion source (ThermoFisher Scientific, Bremen, Germany) and operated under Xcalibur software 2.2 (ThermoFisher Scientific, Bremen, Germany) as described by Kubicova et al. [32]. Theoretical masses and characteristic iron isotopic patterns were calculated by Xcalibur software 2.2 (ThermoFisher Scientific, Bremen, Germany). The mass spectra were recorded for various metal-to-ligand ratios (2:1, 1:1, 1:2 and 1:4).

### 2.3. Differential Pulse Voltammetry

The detailed procedures have been described previously [30]. For records of differential pulse voltammograms, a three-electrode system, µAutolab PGSTAT type III (EcoChemie Inc., Utrecht, The Netherlands), was used. A glassy carbon electrode (3 mm in diameter) served as a working electrode, a platinum wire as a counter electrode, and Ag/AgCl (3 M aqueous solution of KCl) as a reference electrode. The electrochemical experiments were performed using the following parameters: The effective scan rate of the voltammetry was set to 21 mV/s, modulation time to 0.05 s, modulation amplitude to 25 mV, and the scan potential was from −0.500 to +1.350 V. The solution of supporting electrolyte was degassed buffer (0.1 M phosphate buffer pH 7.4; buffer ionic strength 1 M, adjusted by K_2_SO_4_). The measuring atmosphere was argon. Several metal-to-ligand ratios were analyzed (1:1, 1:2, 1:3 and 1:4).

### 2.4. Deoxyribose Degradation Assay

The procedures of deoxyribose degradation assay were performed as described in detail elsewhere [33]. The used buffer was aqueous solutions of KH_2_PO_4_/KOH (30 mM, pH 7.4). The assay is carried out in eight variants; four of them without addition of ethylenediaminetetraacetic acid (EDTA) and four of them in the presence of EDTA as Fe chelator. The basic assay variants are represented by H_2_O_2_/FeCl_3_/ascorbic acid or H_2_O_2_/Fe^III^EDTA/ascorbic acid variants, the other assay variants omitted H_2_O_2_ and/or ascorbic acid. The H_2_O_2_/FeCl_3_/ascorbic acid or H_2_O_2_/Fe^III^EDTA/ascorbic acid reaction mixtures served as the positive control, represented 100% thiobarbituric acid reactive species (TBARS) detection in all variants and also served as the comparative standard for each experiment. Blanks contained the full reaction mixtures, except for 2-deoxyribose, and were determined in each experiment. Experiments were performed in triplicate. The temperature during incubation was 27 °C. Variants containing H_2_O_2_ were evaluated after 1 h; variants without H_2_O_2_ were evaluated after 16 h of incubation.

### 2.5. Fe^II^ Autoxidation Assay

The procedures and reaction mechanisms were published by Chobot et al. [34]. The aqueous solutions of KH_2_PO_4_/KOH (30 mM, pH 7.4) were used as the buffer. The reaction mixtures contained a test substance diluted serially, 2-deoxyribose and FeSO_4._ Blanks contained the full reaction mixtures, except for 2-deoxyribose. Reaction mixtures were incubated at 27 °C for 16 h. Thiobarbituric acid reactive species (TBARS) were determined photometrically with a microplate reader (Tecan Infinite M200, Männedorf, Switzerland) at 532 nm. Experiments were performed in triplicate. Reaction mixtures lacking the test compound served as the positive control (100% TBARS).

## 3. Results

### 3.1. Mass Spectrometry

**Figure 2:**
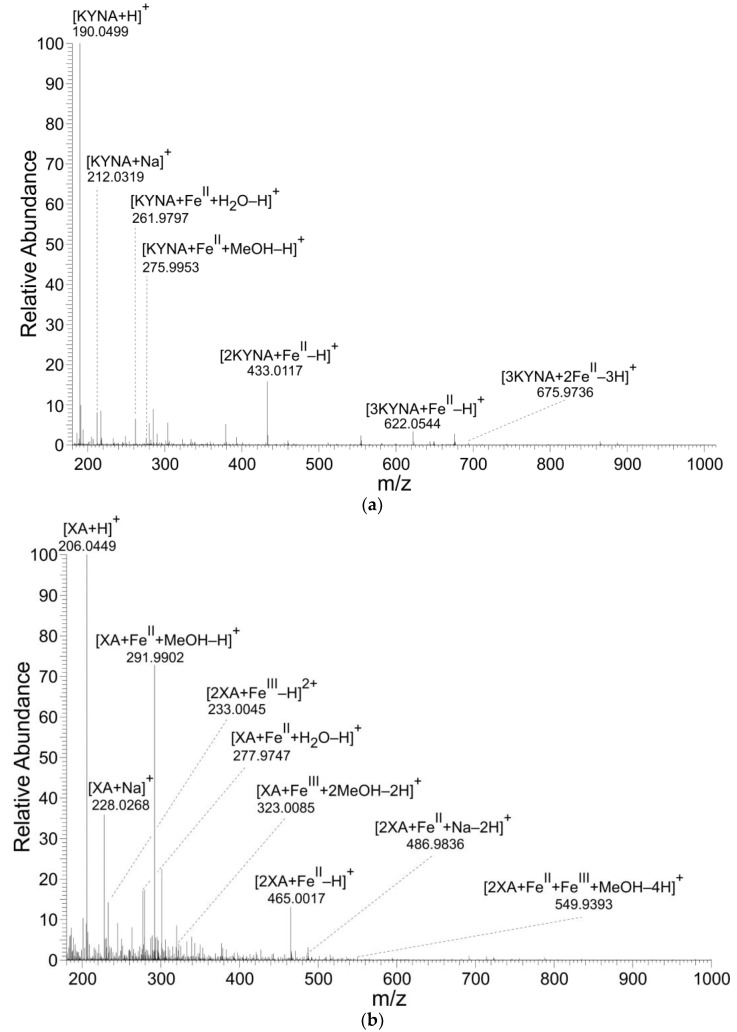

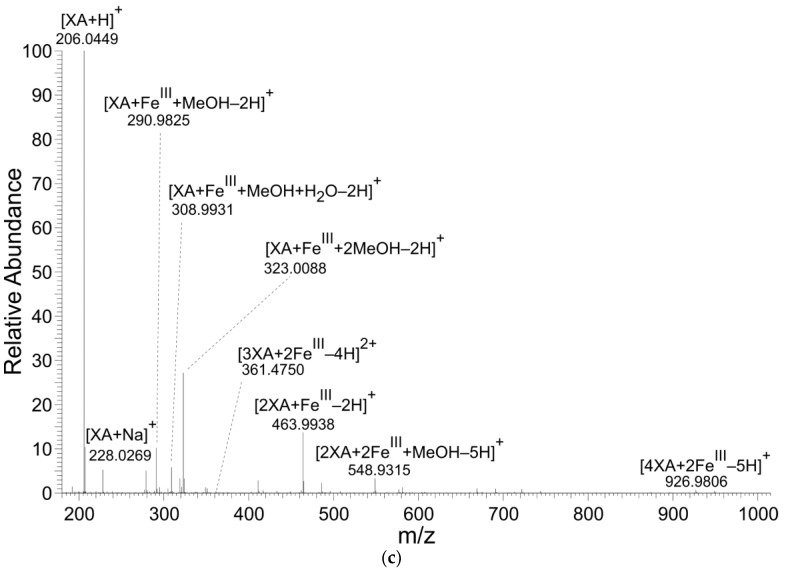
Mass spectra of coordination complexes in solutions of (**a**) Fe^II^ with KYNA, (**b**) Fe^II^ with XA and (**c**) Fe^III^ with XA, detected by nano-ESI‒MS, positive ionization mode. The solutions were prepared by mixing of the KYNA or XA solutions with Fe^II^ or Fe^III^ ions solutions in a molar ratio of metal to ligand of 1:2.

KYNA and XA formed coordination complexes with Fe^II^ ions (Table 1, Table 2 and Table 3 and Figure 2). However, only XA entered coordination complexes with Fe^III^ ions. The figures and tables present the results of reaction solutions with the adjusted molar ratio of ligand to Fe ions of 2:1 and assumed *m*/*z* values for coordination complexes of the isotope ^56^Fe. The typical iron isotopic pattern (^54^Fe 5.8%, ^56^Fe 91.7%, ^57^Fe 2.2%, and ^58^Fe 0.3%) corresponded with the pattern of identified coordination complexes.

### 3.2. Differential Pulse Voltammetry

**Figure 3:**
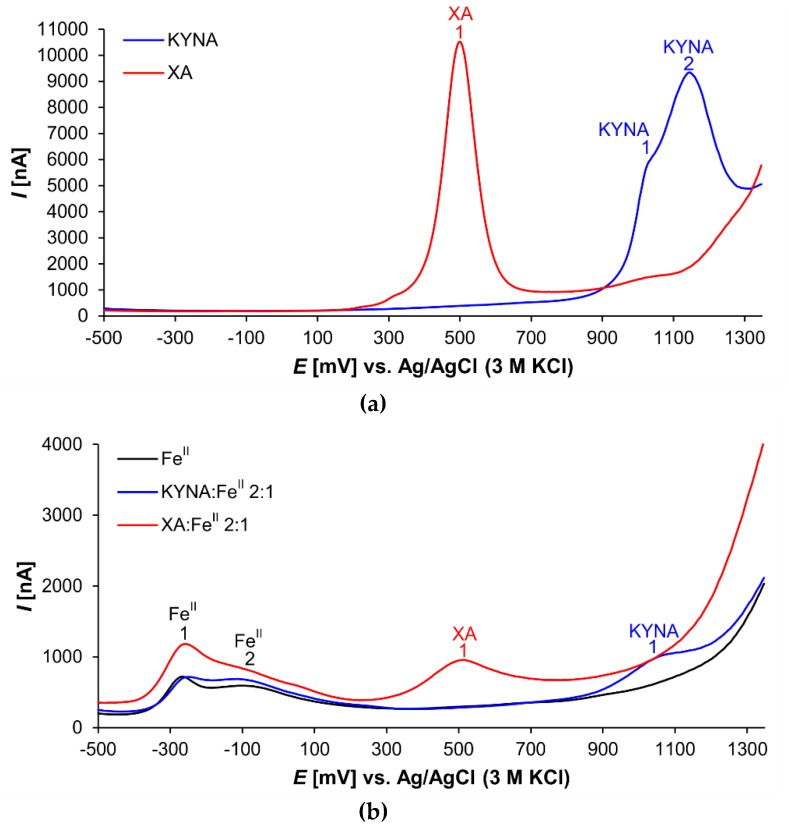
Differential pulse voltammograms of (**a**) KYNA or XA solutions and (**b**) solutions of Fe^II^, 2:1 KYNA:Fe^II^ or 2:1 XA:Fe^II^ mixtures.

Both investigated acids proved electroactive (Figure 3). The XA voltammogram shows one prominent peak (XA 1) at the electrochemical potential of 501 mV (Figure 3a). This signal corresponded probably with 8-hydroxy group oxidation [35].

In the voltammogram of KYNA, two peaks are visible at electrochemical potentials 1020 mV (peak KYNA 1) and 1145 mV (peak KYNA 2), respectively (Figure 3a). Peak KYNA 1 is relatively small and appears as a shoulder of the larger peak KYNA 2 that was a signal of 4-hydroxy group oxidation [36]. Nevertheless, the shapes of the oxidation peaks of both explored acids, KYNA and XA, indicate the complexity of the redox reactions. With high probability, the electrochemical reactions of the phenolic groups were followed by further chemical reactions [37].

Figure 3b illustrates the voltammetric curves of pure Fe^II^ solution, and mixture solutions of KYNA or XA with Fe^II^ (ligand:metal 2:1 ratio). The Fe^II^ curve shows two signals (peak Fe^II^ 1 at −261 mV and peak Fe^II^ 2 at −69 mV). Additional weak peaks and shoulders were caused by mixtures of Fe^II^ coordination complexes with phosphate anions and other components of the buffer as ligands.

The voltammogram of the XA:Fe^II^ mixture solution exhibits the prominent apparently tailing peak Fe^II^ 1. Furthermore, the peak XA 1 corresponding to the 8-hydroxy group oxidation is evidently smaller compared to the peak of free XA.

The voltammogram of the KYNA:Fe^II^ mixture solution differs dramatically from the curve of the free KYNA. The signal of 4-hydroxy group redox reaction at 1145 mV (Figure 3, peak KYNA 2) almost disappears. In the voltammogram is saved only the small signal at 1062 mV, which is slightly shifted to the anodic direction in comparison to the free KYNA (Figure 3, peak KYNA 1 at 1020 mV). Furthermore, the peak of Fe^II^ shows one broad tailing signal.

### 3.3. Deoxyribose Degradation Assay

The deoxyribose degradation assay investigates possible interactions of test substances with hydroxyl radical production in an iron catalyzed Fenton-like reaction. The reaction mixture contained test substance, H_2_O_2_, Fe^III^, ascorbic acid and 2-deoxyribose. The oxidation of 2-deoxyribose led to the production of thiobarbituric acid reactive species (TBARS). Ascorbic acid, a reducing agent, started the Fenton reaction by reduction of Fe^III^ to Fe^II^. Iron was added either as a coordination complex with ethylenediaminetetraacetic acid (EDTA) or as FeCl_3_, which can form complexes with the test compound. EDTA prevents coordination complex formation with a test substance as a ligand. The variants of deoxyribose degradation assay with presence of H_2_O_2_ simulated a situation of tissue damage with high ROS concentrations. The assay variants, where H_2_O_2_ and/or ascorbic acid were omitted, provided more extensive information about the redox chemistry of the test compounds [33].

**Figure 4:**
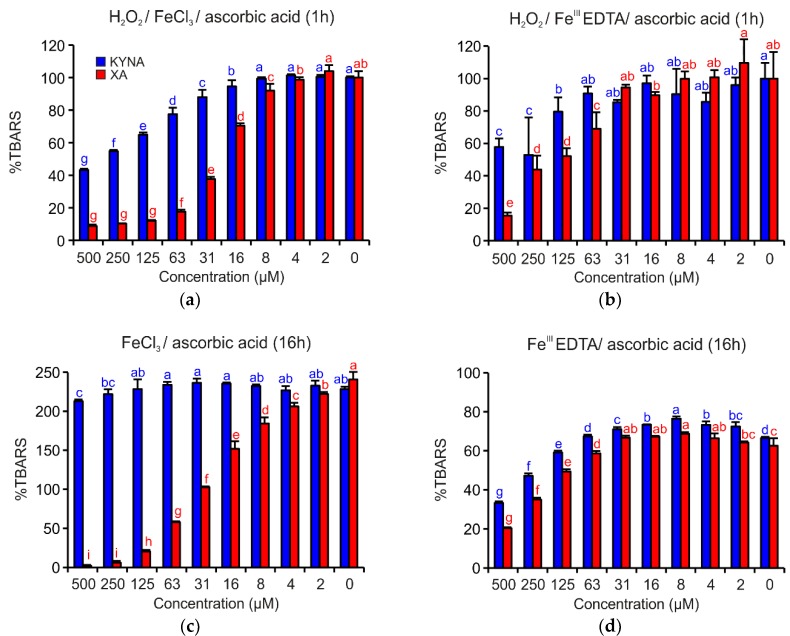
Inhibition effects of KYNA or XA on TBARS formation in the deoxyribose degradation assay: (**a**) H_2_O_2_/FeCl_3_/ascorbic acid, (**b**) H_2_O_2_/Fe^III^EDTA/ascorbic acid, (**c**) FeCl_3_/ascorbic acid and (**d**) Fe^III^EDTA/ascorbic acid. The bars represent the mean of three replications (±S.D.). Letters above the bars indicate significance levels (ANOVA with 95% Duncan’s post hoc test). TBARS: thiobarbituric acid reactive species, S.D.: standard deviation.

Figure 4 shows that both acids demonstrated apparent antioxidant effects. However, in all investigated reaction mixtures, XA was a more efficient antioxidant than KYNA. In the reaction mixture containing H_2_O_2_/FeCl_3_/ascorbic acid and H_2_O_2_/Fe^III^EDTA/ascorbic acid, the antioxidant activities were evident up to the concentration 16 µM or 125 µM, respectively (Figure 4a,b). Coordination complex formation with either of the two quinoline acids enhanced antioxidant activity.

In the reaction mixtures missing H_2_O_2_, such as FeCl_3_/ascorbic acid or Fe^III^EDTA/ascorbic acid (Figure 4c,d), XA inhibited TBARS generation, a type of 2-deoxyribose degradation products, more effectively than KYNA. The different antioxidant activities of both acids are especially visible in the case of reaction mixture FeCl_3_/ascorbic acid (Figure 4c), when KYNA could not prevent the oxidative degradation of 2-deoxyribose with the exception of the two highest tested concentrations.

In the other variants of the deoxyribose degradation assay such as H_2_O_2_/FeCl_3_, H_2_O_2_/Fe^III^EDTA, FeCl_3_, and Fe^III^EDTA, no significant activities were observed (data not shown).

### 3.4. Fe^II^ Autoxidation Assay

This assay extended the insights of deoxyribose degradation assay in terms of the capability of the test compound as ligand of Fe^II^ central atom to scavenge or induce ROS during iron autoxidation and to affect Fe^II^/Fe^III^ redox cycling. In this assay, iron did not need to be reduced before participation in the Fenton reaction. Molecular oxygen that diffuses into reaction mixtures was reduced by Fe^II^ to superoxide anion radical, which can spontaneously dismutate or be reduced to hydrogen peroxide. Then, hydrogen peroxide started the Fenton reaction and hydroxyl radical production. Hydroxyl radicals were detected as TBARS (2-deoxyribose decomposition products) [34].

**Figure 5:**
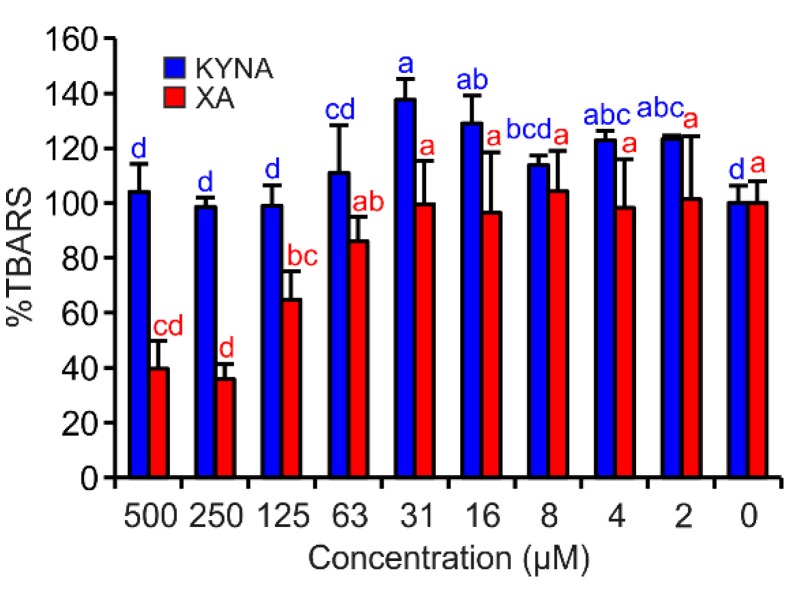
KYNA or XA effects on TBARS production in Fe^II^ autoxidation assay. The bars are the means of three replications (±S.D.). Letters above the bars indicate significance levels (ANOVA with 95% Duncan’s post hoc test). TBARS: thiobarbituric acid reactive species, S.D.: standard deviation.

In this assay, only XA exhibited significant antioxidant effects. By contrast, KYNA showed very weak pro-oxidant properties in the test concentrations of 16 and 31 µM (Figure 5).

## 4. Discussion

KYNA and XA represent endogenous redox active metabolites that can cause either pro- or antioxidant effects [5]. This specifically observed capability is determined by complex combinatory effects of ROS scavenging activities and inhibition of iron redox cycling, the latter of which is important for the ROS generation, especially the strongly oxidant hydroxyl radical, a product of the iron-catalysed Fenton reaction [38].

The mass spectrometry results corroborated KYNA’s and XA’s formation of Fe ion coordination complexes. However, only XA was liganded by both Fe^II^ and Fe^III^ forming mono or binuclear coordination complexes. In these binuclear coordination complexes, Fe was present either in the same or different valency stages. KYNA coordinated Fe^II^ ions, also in mononuclear and binuclear complexes (Table 1, Figure 3a). However, no coordination complexes of KYNA with trivalent Fe ions were detected. Contrary to our results, Minakata et al. failed to detect Fe−KYNA coordination complexes spectrophotometrically [26].

The differential pulse voltammetry investigation corresponds the mass spectrometry results. The 8-hydroxy group present in XA substantially improves Fe liganding. The intensity of the 8-hydroxy group signal varied according to the Fe:XA ratio, a phenomenon that also became evident in a previously published electrochemical study on 8-hydroxyquinoline [39]. However, the peak absence of the KYNA 4-hydroxy group appeared to be independent of the Fe:KYNA concentration ratio.

Additionally, the voltammograms illustrate the anodic shift in the redox potentials of Fe coordination complexes with KYNA or XA in comparison to the free ligands and metal ions. In the presence of iron, the quinolines competed with the components of the phosphate buffer solution to ligand the central atom. Accordingly, Fe^II^ atoms coordinating either KYNA or XA are more difficult to oxidize, whereas reduction of Fe^III^ atoms in coordination complexes is easier.

The DPV (differential pulse voltammetry) results on the one hand, and the deoxyribose degradation and Fe^II^ autoxidation assay results on the other hand, agree. In both assays, XA behaved as a potent antioxidant (Figure 4 and Figure 5). These results suggest that XA’s antioxidant effects depend on its ability to form coordination complexes with Fe ions (deoxyribose assay variants without EDTA, Figure 4a,c and Fe^II^ autoxidation assay, Figure 5) and additionally on scavenging hydroxyl radicals (deoxyribose assay variants with EDTA, Figure 4b,d). Comparing XA with 8-hydroxyquinoline in terms of antioxidant activity in similar assays, XA was less efficient [39,40]. However, in contrast to the methodological approach of this study, Murakami et al. observed the pro-oxidant effects of XA [29]. Additionally, the results of this study point to the important contribution of the 8-hydroxy group to more pronounced antioxidant properties of XA. Moreover, the ROS scavenging effect of XA is supported by the conjugated and aromatic system that stabilizes the formed semiquinone radical [41].

An in vivo antioxidant study of XA proved that XA played a crucial role in tissue protection against Fe-induced cell death in female midguts of *Aedes aegypti* [42]. Furthermore, XA decreased Fe- or heme-catalysed lipid peroxidation and demonstrated a strong contribution of coordination complex formation with Fe^II^ ions to alleviating oxidative damage. A previous study supports the present observations by also reporting the mitigating effects of XA on metal-catalysed lipid peroxidation [27]. Additionally and in accordance, a mosquito strain, which is only able to produce KYNA in its midgut, proved to be less efficiently protected against Fe-induced tissue damage [42].

Compared to XA, KYNA proved to be not antioxidant in the FeCl_3_/ascorbic acid deoxyribose degradation assay variant (Figure 4c) and showed only negligible activities in Fe^II^ autoxidation assay (Figure 5). In both cases, KYNA had the opportunity to form coordination complexes with Fe ions. KYNA was not able to prevent the reduction of molecular oxygen to superoxide anion radical and consequent TBARS generation. It is possible that Fe−KYNA coordination complexes increase the dismutation rate of superoxide anion radical to hydrogen peroxide. Mahal et al. reported comparable catalytic effects on superoxide anion radical dismutation of Fe coordination complexes with phenols and aminophenols [43]. These observations also correspond well to those reported by Murakami et al., who observed no effects of KYNA on Fe^II^ autoxidation-induced lipid peroxidation [27]. Probably, KYNA inhibits 2-deoxyribose oxidative degradation primarily by hydroxyl radical scavenging, which corresponds to the effects that are visible in the full reaction mixture of the deoxyribose degradation assay (Figure 4a,b). Lugo-Lutron et al., who investigated KYNA antioxidant properties by a set of assays, including the H_2_O_2_/Fe^III^EDTA/ascorbic acid variant of deoxyribose degradation assay, obtained similar results [25]. In other types of antioxidant assays, KYNA showed no or only weak antioxidant activity [25,26]. Generally, KYNA acts as an antioxidant in higher concentrations than XA. However, some KYNA chemical derivatives are more efficient in passing the BBB and become metabolized to KYNA afterwards [44].

In the deoxyribose degradation assay variants, where ascorbic acid was omitted, neither KYNA nor XA was able to substitute ascorbic acid function and to reduce Fe^III^ to Fe^II^. Thus, consequently, the Fenton reaction could not be started. Therefore, no activities were observed in these four deoxyribose degradation assay variants (data not shown).

**Figure 6:**
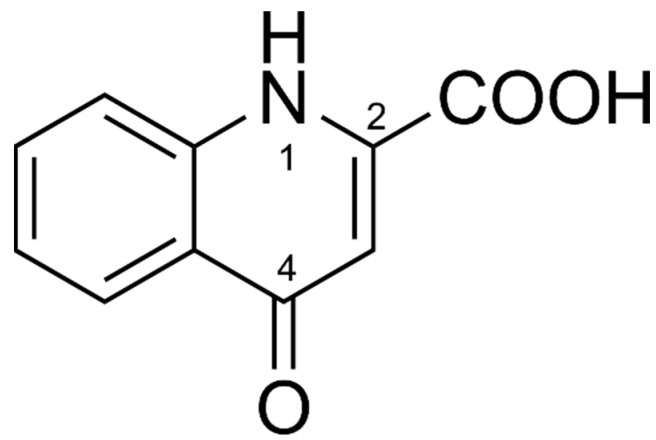
Chemical structure of KYNA keto tautomer.

The DPV of Fe−KYNA coordination complexes lack an oxidation peak of the 4-hydroxy group. A possible reason for this may be the formation of the more stable KYNA keto tautomer (Figure 6) in the coordination complexes with Fe^II^. The peak KYNA 1 in the DPV probably corresponds to the oxidation of heterocyclic NH group at position 1.

## 5. Conclusions

The chemical properties of KYNA and XA provide evidence that both acids can affect brain tissues by interactions with various receptors and further by pre-receptor chemistry. They contribute to the maintenance of redox and iron homeodynamics when mitochondria become damaged and the permeability of the blood‒brain barrier is increased. Damaged mitochondria and the blood–brain barrier are often associated with neurodegenerative diseases. Therefore, modulation of KYNA and XA concentrations in the brain represent a promising therapeutic approach to retard the cognitive disfunction development of patients that suffer from Alzheimer’s or Parkinson’s diseases.

## Figures and Tables

**Table 1:** The main signals of ^56^Fe‒KYNA coordination complexes in the solutions of KYNA with Fe^II^ analyzed by nano-ESI‒MS; a positive ionization mode.

Composition	Formula	m/z Calculated	m/z Found	∆ [ppm]
[KYNA+H]^+^	[C_10_H_8_NO_3_]^+^	190.0499	190.0499	0.13
[KYNA+Na]^+^	[C_10_H_7_NNaO_3_]^+^	212.0318	212.0319	0.42
[KYNA+Fe^II^+H_2_O-H]^+^	[C_10_H_8_FeNO_4_]^+^	261.9797	261.9797	0.01
[KYNA+Fe^II^+MeOH-H]^+^	[C_11_H_10_FeNO_4_]^+^	275.9954	275.9953	−0.10
[2KYNA+Fe^II^-H]^+^	[C_20_H_13_FeN_2_O_6_]^+^	433.0118	433.0117	−0.04
[3KYNA+Fe^II^-H]^+^	[C_30_H_20_FeN_3_O_9_]^+^	622.0543	622.0544	0.02
[3KYNA+2Fe^II^-3H]^+^	[C_30_H_18_Fe_2_N_3_O_9_]^+^	675.9736	675.9736	−0.07

**Table 2:** The main signals of ^56^Fe‒XA coordination complexes in the solutions of XA with Fe^II^ analyzed by nano-ESI‒MS; a positive ionization mode.

Composition	Formula	m/z Calculated	m/z Found	∆ [ppm]
[XA+H]^+^	[C_10_H_8_NO_4_]^+^	206.0448	206.0449	0.45
[XA+Na]^+^	[C_10_H_7_NNaO_4_]^+^	228.0267	228.0268	0.41
[2XA+Fe^III^-H]^2+^	[C_20_H_14_FeN_2_O_8_]^2+^	233.0044	233.0045	0.18
[XA+Fe^II^+H_2_O-H]^+^	[C_10_H_8_FeNO_5_]^+^	277.9746	277.9747	0.08
[XA+Fe^II^+MeOH-H]^+^	[C_11_H_10_FeNO_5_]^+^	291.9903	291.9902	−0.34
[XA+Fe^III^+2MeOH-2H]^+^	[C_12_H_13_FeNO_6_]^+^	323.0087	323.0085	−0.48
[2XA+Fe^II^-H]^+^	[C_20_H_13_FeN_2_O_8_]^+^	465.0016	465.0017	0.16
[2XA+Fe^II^+Na-2H]^+^	[C_20_H_12_FeN_2_NaO_8_]^+^	486.9835	486.9836	0.23
[2XA+Fe^II^+Fe^III^+MeOH-4H]^+^	[C_21_H_14_Fe_2_N_2_O_9_]^+^	549.9393	549.9393	0.10

**Table 3:** The main signals of ^56^Fe‒XA coordination complexes in the solutions of XA with Fe^III^ analyzed by nano-ESI‒MS; a positive ionization mode.

Composition	Formula	m/z Calculated	m/z Found	∆ [ppm]
[XA+H]^+^	[C_10_H_8_NO_4_]^+^	206.0448	206.0449	0.45
[XA+Na]^+^	[C_10_H_7_NNaO_4_]^+^	228.0267	228.0269	0.82
[XA+Fe^III^+MeOH-2H]^+^	[C_11_H_9_FeNO_5_]^+^	290.9825	290.9825	−0.05
[XA+Fe^III^+MeOH+H_2_O-2H]^+^	[C_11_H_11_FeNO_6_]^+^	308.9930	308.9931	0.16
[XA+Fe^III^+2MeOH-2H]^+^	[C_12_H_13_FeNO_6_]^+^	323.0087	323.0088	0.24
[3XA+2Fe^III^-4H]^2+^	[C_30_H_17_O_12_N_3_Fe_2_]^2+^	361.4750	361.4750	−0.01
[2XA+Fe^III^-2H]^+^	[C_20_H_12_FeN_2_O_8_]^+^	463.9938	463.9938	0.16
[2XA+2Fe^III^+MeOH-5H]^+^	[C_21_H_13_Fe_2_N_2_O_9_]^+^	548.9314	548.9315	0.15
[4XA+2Fe^III^-5H]^+^	[C_40_H_23_Fe_2_N_4_O_16_]^+^	926.9802	926.9806	0.35

## Abbreviations

BBB	Blood−brain barrier
DNA	Deoxyribonucleic acid
DPV	Differential pulse voltammetry
EDTA	Ethylenediaminetetraacetic acid
KYNA	Kynurenic acid
Nano-ESI−MS	Nano-electrospray−mass spectrometry
ROS	Reactive oxygen species
S.D.	Standard deviation
TBARS	Thiobarbituric acid reactive species
XA	Xanthurenic acid

## References

[1] Graves D.B. (2012). The emerging role of reactive oxygen and nitrogen species in redox biology and some implications for plasma applications to medicine and biology. J. Phys. D Appl. Phys..

[2] Nordzieke D.E., Medraño-Fernandez I. (2018). The plasma membrane: A platform for intra- and intercellular redox signaling. Antioxidants.

[3] Hörandl E., Hadacek F. (2013). The oxidative damage initiation hypothesis for meiosis. Plant Reprod..

[4] Hadacek F., Bachmann G. (2015). Low-molecular-weight metabolite systems chemistry. Front. Environ. Sci..

[5] Esquivel D.G., Ramirez-Ortega D., Pineda B., Castro N., Rios C., de la Cruz V. (2017). Kynurenine pathway metabolites and enzymes involved in redox reactions. Neuropharmacology.

[6] Schwarcz R., Stone T.W. (2017). The kynurenine pathway and the brain: Challenges, controversies and promises. Neuropharmacology.

[7] Erhardt S., Schwieler L., Imbeault S., Engberg G. (2017). The kynurenine pathway in schizophrenia and bipolar disorder. Neuropharmacology.

[8] Lovelace M.D., Varney B., Sundaram G., Lennon M.J., Lim C.K., Jacobs K., Guillemin G.J., Brew B.J. (2017). Recent evidence for an expanded role of the kynurenine pathway of tryptophan metabolism in neurological diseases. Neuropharmacology.

[9] Wirthgen E., Hoeflich A., Rebl A., Gunther J. (2017). Kynurenic acid: The Janus-faced role of an immunomodulatory tryptophan metabolite and its link to pathological conditions. Front. Immunol..

[10] Fazio F., Lionetto L., Curto M., Iacovelli L., Copeland C.S., Neale S.A., Bruno V., Battaglia G., Salt T.E., Nicoletti F. (2017). Cinnabarinic acid and xanthurenic acid: Two kynurenine metabolites that interact with metabotropic glutamate receptors. Neuropharmacology.

[11] Tan L., Yu J.T. (2012). The kynurenine pathway in neurodegenerative diseases: Mechanistic and therapeutic considerations. J. Neurol. Sci..

[12] Majewski M., Kozlowska A., Thoene M., Lepiarczyk E., Grzegorzewski W.J. (2016). Overview of the role of vitamins and minerals on the kynurenine pathway in health and disease. J. Physiol. Pharmacol..

[13] Colin-Gonzalez A.L., Maldonado P.D., Santamaria A. (2013). 3-Hydroxykynurenine: An intriguing molecule exerting dual actions in the central nervous system. Neurotoxicology.

[14] Moroni F., Russi P., Lombardi G., Beni M., Carla V. (1988). Presence of kynurenic acid in the mammalian brain. J. Neurochem..

[15] Moroni F., Cozzi A., Sili M., Mannaioni G. (2012). Kynurenic acid: A metabolite with multiple actions and multiple targets in brain and periphery. J. Neural. Transm..

[16] Turski M.P., Kaminski P., Zgrajka W., Turska M., Turski W.A. (2012). Potato- An important source of nutritional kynurenic acid. Plant Food Hum. Nutr..

[17] Dehhaghi M., Kazemi Shariat Panahi H., Guillemin G.J. (2019). Microorganisms, tryptophan metabolism, and kynurenine pathway: A complex interconnected loop influencing human health status. Int. J. Tryptophan. Res..

[18] Kennedy P.J., Cryan J.F., Dinan T.G., Clarke G. (2017). Kynurenine pathway metabolism and the microbiota-gut-brain axis. Neuropharmacology.

[19] Datki Z., Galik-Olah Z., Bohar Z., Zadori D., Fulop F., Szatmari I., Galik B., Kalman J., Vecsei L. (2019). Kynurenic acid and its analogs are beneficial physiologic attenuators in bdelloid rotifers. Molecules.

[20] Montagne A., Zhao Z., Zlokovic B.V. (2017). Alzheimer’s disease: A matter of blood–brain barrier dysfunction?. J. Exp. Med..

[21] Pretorius L., Kell D.B., Pretorius E. (2018). Iron dysregulation and dormant microbes as causative agents for impaired blood rheology and pathological clotting in Alzheimer’s type dementia. Front. Neurosci..

[22] Kell D.B. (2009). Iron behaving badly: Inappropriate iron chelation as a major contributor to the aetiology of vascular and other progressive inflammatory and degenerative diseases. BMC Med. Genom..

[23] Kell D.B. (2010). Towards a unifying, systems biology understanding of large-scale cellular death and destruction caused by poorly liganded iron: Parkinson’s, Huntington’s, Alzheimer’s, prions, bactericides, chemical toxicology and others as examples. Arch. Toxicol..

[24] Lovell M.A., Robertson J.D., Teesdale W.J., Campbell J.L., Markesbery W.R. (1998). Copper, iron and zinc in Alzheimer’s disease senile plaques. J. Neurol. Sci..

[25] Lugo-Huitron R., Blanco-Ayala T., Ugalde-Muniz P., Carrillo-Mora P., Pedraza-Chaverri J., Silva-Adaya D., Maldonado P.D., Torres I., Pinzon E., Ortiz-Islas E. (2011). On the antioxidant properties of kynurenic acid: Free radical scavenging activity and inhibition of oxidative stress. Neurotoxicol. Teratol..

[26] Minakata K., Fukushima K., Nakamura M., Iwahashi H. (2011). Effect of some naturally occurring iron ion chelators on the formation of radicals in the reaction mixtures of rat liver microsomes with ADP, Fe and NADPH. J. Clin. Biochem. Nutr..

[27] Murakami K., Ito M., Yoshino M. (2001). Xanthurenic acid inhibits metal ion-induced lipid peroxidation and protects NADP-isocitrate dehydrogenase from oxidative inactivation. J. Nutr. Sci. Vitaminol..

[28] Lopez-Burillo S., Tan D.X., Mayo J.C., Sainz R.M., Manchester L.C., Reiter R.J. (2003). Melatonin, xanthurenic acid, resveratrol, EGCG, vitamin C and α-lipoic acid differentially reduce oxidative DNA damage induced by Fenton reagents: A study of their individual and synergistic actions. J. Pineal Res..

[29] Murakami K., Haneda M., Yoshino M. (2006). Prooxidant action of xanthurenic acid and quinoline compounds: Role of transition metals in the generation of reactive oxygen species and enhanced formation of 8-hydroxy-2’-deoxyguanosine in DNA. Biometals.

[30] Chobot V., Hadacek F., Weckwerth W., Kubicova L. (2015). Iron chelation and redox chemistry of anthranilic acid and 3-hydroxyanthranilic acid: A comparison of two structurally related kynurenine pathway metabolites to obtain improved insights into their potential role in neurological disease development. J. Organomet. Chem..

[31] Kubicova L., Hadacek F., Chobot V. (2013). Quinolinic Acid: Neurotoxin or oxidative stress modulator?. Int. J. Mol. Sci..

[32] Kubicova L., Hadacek F., Weckwerth W., Chobot V. (2015). Effects of endogenous neurotoxin quinolinic acid on reactive oxygen species production by Fenton reaction catalyzed by iron or copper. J. Organomet. Chem..

[33] Chobot V. (2010). Simultaneous detection of pro- and antioxidative effects in the variants of the deoxyribose degradation assay. J. Agric. Food Chem..

[34] Chobot V., Hadacek F., Kubicova L. (2014). Effects of selected dietary secondary metabolites on reactive oxygen species production caused by iron(II) autoxidation. Molecules.

[35] Stevic M.C., Ignjatovic L.M., Ciric-Marjanovic G., Stanisic S.M., Stankovic D.M., Zima J. (2011). Voltammetric behaviour and determination of 8-hydroxyquinoline using a glassy carbon paste electrode and the theoretical study of its electrochemical oxidation mechanism. Int. J. Electrochem. Sci..

[36] Giles G.I., Collins C.A., Stone T.W., Jacob C. (2003). Electrochemical and in vitro evaluation of the redox-properties of kynurenine species. Biochem. Biophys. Res. Commun..

[37] Bard A.J., Faulkner L.R. (2001). Electrochemical Methods: Fundamentals and Applications.

[38] Koppenol W.H., Hider R.H. (2019). Iron and redox cycling. Do’s and don’ts. Free Radical Biol. Med..

[39] Chobot V., Hadacek F., Bachmann G., Weckwerth W., Kubicova L. (2018). Antioxidant properties and the formation of iron coordination complexes of 8-hydroxyquinoline. Int. J. Mol. Sci..

[40] Chobot V., Drage S., Hadacek F. (2011). Redox properties of 8-quinolinol and implications for its mode of action. Nat. Prod. Commun..

[41] Rice-Evans C.A., Miller N.J., Paganga G. (1996). Structure-antioxidant activity relationships of flavonoids and phenolic acids. Free Radical Biol. Med..

[42] Lima V.L.A., Dias F., Nunes R.D., Pereira L.O., Santos T.S.R., Chiarini L.B., Ramos T.D., Silva-Mendes B.J., Perales J., Valente R.H. (2012). The antioxidant role of xanthurenic acid in the Aedes aegypti midgut during digestion of a blood meal. PLoS ONE.

[43] Mahal H.S., Kapoor S., Satpati A.K., Mukherjee T. (2005). Radical scavenging and catalytic activity of metal-phenolic complexes. J. Phys. Chem. B.

[44] Stone T.W. (2000). Development and therapeutic potential of kynurenic acid and kynurenine derivatives for neuroprotection. Trends Pharmacol. Sci..

